# Delaunay Triangulation as a New Coverage Measurement Method in Wireless Sensor Network

**DOI:** 10.3390/s110303163

**Published:** 2011-03-15

**Authors:** Hassan Chizari, Majid Hosseini, Timothy Poston, Shukor Abd Razak, Abdul Hanan Abdullah

**Affiliations:** 1 Faculty of Computer Science and Information Systems, Universiti Teknologi Malaysia, Malaysia; E-Mails: chizari@ieee.org (H.C.); majid@ieee.org (M.H.); shukorar@utm.my (S.A.R.); 2 Chief Scientist, Nordic River Software AB, Umeå, Sweden; E-Mail: tim.poston@gmail.com

**Keywords:** wireless sensor network, sensing coverage, communication coverage, quality of coverage, delaunay triangulation

## Abstract

Sensing and communication coverage are among the most important trade-offs in Wireless Sensor Network (WSN) design. A minimum bound of sensing coverage is vital in scheduling, target tracking and redeployment phases, as well as providing communication coverage. Some methods measure the coverage as a percentage value, but detailed information has been missing. Two scenarios with equal coverage percentage may not have the same Quality of Coverage (QoC). In this paper, we propose a new coverage measurement method using Delaunay Triangulation (DT). This can provide the value for all coverage measurement tools. Moreover, it categorizes sensors as ‘fat’, ‘healthy’ or ‘thin’ to show the dense, optimal and scattered areas. It can also yield the largest empty area of sensors in the field. Simulation results show that the proposed DT method can achieve accurate coverage information, and provides many tools to compare QoC between different scenarios.

## 1. Introduction

Wireless Sensor Networks (WSNs) have attracted attention in much research on *ad hoc* networks. The many challenges in successful WSN implementation include sensor scheduling, routing, re-deployment and sensor movement. Since the WSN goal is to sense a phenomenon, in each challenge a fit tool to measure sensing coverage is important to success.

Sensing coverage is defined [1] as the ratio of the sensible area to the entire desired area. While in the ideal environment these areas must be equal (Deterministic Coverage), Zhang *et al.* [2] showed that the sacrifice of a small amount of coverage (Stochastic Coverage) can increase network lifetime by 3 to 7 times. A gain in network lifetime is very important in WSN, where it is usually impractical to change or charge the sensors’ batteries, and very costly to deploy new sensors on the field. Stochastic coverage is widely accepted by researchers and WSN application designers. Like sensing coverage, communication coverage is a deterministic factor which needs all the active sensors to be able to communicate with one another. An important issue in any WSN is to check whether the communication coverage is complete among active sensors.

A WSN mission is usually started by deploying a large number of sensors. A scheduling algorithm defines different sets of sensors, which must each achieve a lower bound coverage value set in the mission goal. When one set of sensors is activated, the rest are turned off and wait for their time triggers to be activated. A scheduling algorithm can increase the lifetime of a WSN by reserving the energy in redundant sensors.

One class of scheduling algorithm needs global information about sensors and their positions, while others just work with local information gathered by each sensor about its neighbors. Either way, a good scheduling algorithm that covers the whole network can prolong the network lifetime. However, different methods to measure sensing coverage may give various results, which makes comparison hard. The term of Quality of Coverage (QoC) has not been defined clearly enough to provide a judgment tool between different coverage algorithms.

In this paper, we propose a new method to determine sensing/communication coverage, which provides more detailed QoC information than its predecessors about the uniformity of coverage, which has remarkable influence on network efficiency. This technique, based on Delaunay Triangulation (DT), is useful in many different challenges of WSN.

Organizationally, Section 2 discusses the research background, prior methods for calculating sensing coverage, and some previous research in WSN that used DT. Section 3 introduces the proposed coverage measurement tool. Section 4 provides four methods for analyzing the DT results. The paper concludes with Section 5.

## 2. Research Background

There are several ways to define coverage in WSNs, each with advantages and disadvantages. This section discusses existing calculation methods, and presents other known applications of DT in WSN.

### 2.1. Coverage Calculation Methods

The simplest measure of sensing coverage [3,4] divides the mission field into a grid of small squares, each representing one sensible area that should contain at least one sensor: the exact location of sensors inside the squares is ignored. The sensing coverage is the percentage of squares with at least one active sensor inside.

The favorite definition of sensing and communication coverage is the circular model [2,5–8]. In this model, the sensors have a sensing radius *R_s_*, whose value could be a constant like *R_s_* = 20*m* [5], or related to transmission range (*R_t_*) by $R_{s}>=R_{t}/\sqrt{3}$ [2] or *R_s_* = *R_t_*/2 [8].

The circular model with shadowing [1,9,10] is similar, but has an additional radius *R_u_* for a region outside of the guaranteed sensing area, which is still sensible with some probability *p* > 0. Accordingly, the sensing coverage integrates over all target locations the probability:

- If the object is in *R_s_* range, it will be sensed with probability 1;
- If the object is between *R_s_* and *R_u_*, it will be sensed with probability *p*;
- If the object is out of *R_u_* range, it is not sensed.

Another way to quantify sensing coverage is circular probabilistic model [11–13], which is like the circular model with shadowing effect when *R_s_* = 0. It integrates:

- If the object is in the *R_u_* range, it will be sensed with probability *p*;
- The value of *p* decreases with distance from the sensor.
- If the object is out of the *R_u_* range, it is not sensed.

Soreanu *et al.* [14] give a non-unit-circular model for measuring the sensing coverage, with an elliptical sensing area that the sensors can widen or narrow by using different power levels. These adjustments can significantly improve the network coverage.

Voronoi decomposition [15–18] partitions the points of field into convex ‘area of influence’ polygons around their nearest sensors. All previous work has used this as a clustering system to determine sensor scheduling: coverage was still quantified using the circular model.

To the best of our knowledge, the probabilistic circular and non-unit-circular models, like Voronoi decomposition, are used to determine whether or not a phenomenon can be detected, rather than to quantify the overall coverage of a sensor network. Only the grid-based and the circular models are the only methods so far that are employed to determine how much of the desired area is sensible.

### 2.2. Delaunay Triangulation in WSNs

To quantify the Quality of Coverage (QoC) in the empty spaces between sensors requires a spatial segmentation algorithm whose characteristics reveal the QoC information. Among the choices are the Voronoi algorithm, the Gabriel graph [19] and triangulation methods. Voronoi creates a polygon around each sensor. The Gabriel graph is a subgraph of the Delaunay triangulation edge graph, so its edges divide the plane into larger polygons. A triangulation algorithm creates a graph of edges between sensors, which segment the plane into triangles, where many mathematical procedures are more practical than on polygons with different numbers of vertices.

The Delaunay Triangulation (DT) is a geometrically optimized triangulation. It has many applications in computer science, such as three dimensional (3D) modeling of objects and graph analysis. In WSN, Wu *et al.* [20] used DT to find the largest free space inside a network for the next deployment target; Wang *et al.* [21] found an optimal sensing coverage radius for each sensor for stochastic coverage with reduced energy usage; Vu *et al.* [22] corrected Wang *et al.* [21], with a focus on optimizing sensing radii for border sensors. Moreover, Calinescu [23] used DT to propose a localized routing algorithm.

To calculate DT requires global information: the exact position of all sensors in the network. However, Calinescu [23] proposed a distributed algorithm for an estimated DT, calculated in parallel in all sensors by their local information about their neighbors. Wang *et al.* [21] improved this to make it closer to a DT. Satyanarayana *et al.* [24–26] based localized DT calculation methods on the same concept, applied to *ad hoc* networks. Here we use global information and a classic DT algorithm, but our different analysis methods may also be used in online decision making for sensors with a localized DT algorithm.

## 3. Coverage Measurement Model

The current coverage measurement tools provide the sensible percentage of the desired field *F*, which cannot clarify the uniformity of coverage: how the uncovered areas are distributed. It has been shown [27] that the uniformity of coverage has a great influence on the efficiency of WSN in target tracking. Nittel *et al.* [28] and Ferentinos *et al.* [29] used a Mean Relative Deviation (MRD) formula to grade the uniformity of coverage.

This paper defines Quality of Coverage (QoC) in terms of four concepts: the Probabilistic Distribution Function (PDF) of the distance of each point in the field to its closest sensor (Coverage Resolution Model); uniformity of coverage; the percentage of the sensors in the dense, perfect or scatter areas; and the largest empty space between sensors.

Let *P* be the set of all points in *F*, *S* be the set of all sensors, and ‖*a*, *b*‖ indicate the distance from *a* to *b*. The Coverage Resolution Model (CRM) is defined as a function *C_i_* as follows:

(1) $$\begin{array}{l} P\to \mathbb{R}\\ p\mapsto C_{i}=\underset{s_{j}\in S}{\text{min}}\left(\left\|p,s_{j}\right\|\right) \end{array}$$

showing the distance of closest sensor to each point in the field. This information enables finding the overall coverage value based on circular model, the circular model with shadowing effect, and the circular probabilistic model. It is not an array (since the set *P* is theoretically infinite) but can be approximated by one, using a large set of sample points *p_i_*. The coverage model proposed in this paper can find a good estimate for the CRM as well as other QoC information.

The coverage measurement tool proposed here uses partitioning via triangulation to identify the coverage level in different areas of the field. There are different types of triangulation methods, but an ‘optimized’ one maximizes the minimum angle of each triangle, making it more nearly equilateral. Delaunay Triangulation (DT) is an example of this. A triangulation *T* (*P*) is a Delaunay Triangulation of *P*, denoted as *DT* (*P*), if and only if the circumcircle of any triangle of *T* strictly contains no other point of *P*. For more information about DT algorithms and application, see [30,31].

For a network with more than three sensors, DT is an optimal triangulation with the properties:

1. The outer polygon of the triangulation for a set of points is convex.
2. Each sensor is connected by triangle edges to its closest neighbors.
3. If no three sensors lie on one shared straight line, each has degree at least two.
4. The circumcircle of each triangle contains no other sensor.

**Figure 1.**
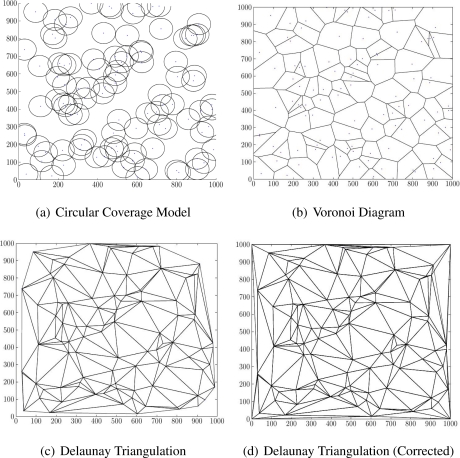
A Sample With Different Coverage Model Approaches.

Several methods [31] can find a DT, such as an incremental algorithm, or divide and conquer. They all need global information (all sensors’ positions), making DT more applicable to global WSN applications. However, local WSN applications can use DT as a benchmark measurement tool, to compare results with other scheduling algorithms. Moreover, current localized DT algorithms, the results of this research could be used for online decision making. Figure 1 shows a random deployment of sensors, with circular coverage model (Figure 1(a)), Voronoi diagram (Figure 1(b)) and DT (Figure 1(c)).

### 3.1. Modification in DT

To use the DT as a WSN coverage measurement tool, we add two rules before generating the DT graph. The first rule adds extra sensors at the corners of the field, assumed convex (in our examples, a square), since as in Figure 1(c), the outer polygon of the coverage model may not cover all the field. Since the outer polygon in DT is always convex, additional sensors on the field corners lead to a full triangulation of the field as in Figure 1(d). Secondly, if three sensors cannot create a triangle because they are collinear, we move one of them by a random multiple of 0.5 m to let the DT create a triangle.

## 4. Analysis Methods

This paper proposes five QoC parameters to analyze the DT. The first step finds local and global communication coverage of a network. The second divides sensors into three categories: in dense, scattered or perfect areas. The third extracts information from DT which easily shows the coverage values for circular model with and without shadowing effect and probability, as well as uniformity of coverage. The last finds the biggest empty area between sensors as a comparison parameter among network planning applications.

### 4.1. Network Coverage Analysis

The term ‘network coverage’ is used for both sensing and communication. Communication network coverage is the ability to send and receive packets to and from all the active sensors in the field. When each sensor has at least one neighbor in its communication range, local communication coverage is satisfied. When a sensor can send information to all active sensors in the field, via other sensors, general communication coverage is achieved. Both local and global communication coverage are very important for a WSN. Network Coverage Analysis (NCA) is a good tool to examine both.

**Figure 2.**
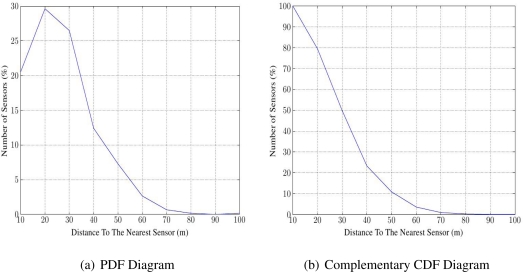
The Nearest Neighbor Distance.

To test local communication coverage, we use the Probabilistic Distribution Function (PDF) of just the closest neighbor for each sensor, as provided by DT. This shows among other things, how many sensors are completely alone, with no neighbor close enough. Figure 2(a) shows a distribution histogram of nearest neighbors, for a random placement network of 600 sensors in a 1,000 m × 1,000 m field. The largest nearest-neighbor distance is about 100 m, while a majority of sensors are at 20 to 30 m from their nearest neighbors. The complementary Cumulative Distribution Function (CDF) plot (Figure 2(b)) shows that about 10% of sensors are more than 50 m from any neighbor. This information is clearly useful in examining communication coverage. For instance, if the communication range for sensors is 50 m, then 10% of sensors have no neighbor in contact, and the local communication coverage is 90%.

**Figure 3.**
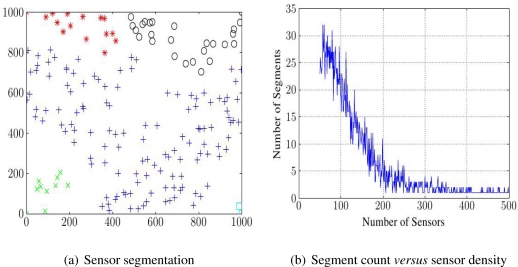
Global Communication Coverage.

Global communication coverage is the ability of each sensor to send information to all others. If a network fails this, its sensors divide into multiple intra-communicating segments, between which no message can pass. NCA must check the global coverage, and the number of such segments. This may be done by a spanning tree algorithm, on the graph of DT with triangle edges longer than transmission range deleted. For example, Figure 3(a) shows global communication segmentation on a 1,000 m × 1,000 m field where *N* = 180 sensors with 100 m communication range are randomly deployed. Figure 3(b) shows how increasing *N* can reduce the number of communication segments.

### 4.2. Sensor State Analysis

A WSN usually starts by deploying many sensors in the mission field, but activating them all wastes their energy as their coverage areas may have large overlaps. To avoid this problem, a scheduling pattern turns some off. The overall coverage value can test the scheduling pattern, but further information may help to explore the weaknesses of the scheduling algorithms.

Sensor State Analysis (SSA) is based on the number of close neighbors. Using this information, we categorize sensors in three groups:

- Sensors with many close neighbors (Fat Sensors)
- Sensors with enough close neighbors (Healthy Sensors)
- Sensors with few close neighbors (Thin Sensors)

**Figure 4.**
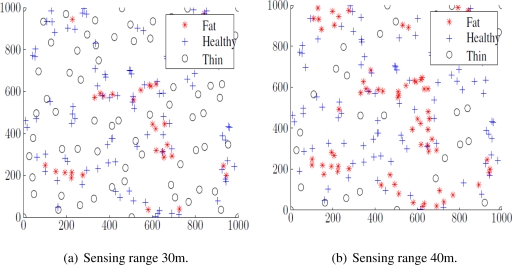
Fat, Healthy and Thin Sensors.

The best thresholds for the close neighbor count in distinguishing these may depend on the application, but as the square and hexagonal sensor placements have ideal coverage, we count a sensor with 4 to 6 close neighbors as a healthy node. More than 6 indicates a fat sensor, and fewer than 4 thin one. A high fat sensor count shows that some sensors in that area could be moved or turned off to save energy. A high thin sensor count indicates uncovered areas, and some redeployment or re-activation is needed to ensure coverage. Figure 4 color-codes the Fat, Healthy and Thin sensors for sensing coverage in a 1,000 m × 1,000 m field with 30 m sensing range Figure 4(a) or 40 m range Figure 4(b). Sensors in dense and non-dense environments are easily recognizable.

### 4.3. Coverage Resolution Analysis

The next information retrieved from DT is Coverage Resolution Analysis (CRA), whose aim is a good estimate for CRM. Any three mutual neighbors *S*_1_, *S*_2_ and *S*_3_ create a triangle *T*. The farthest point from them inside *T* is the center *C* of its circumcircle. Let *d_s_* be the distance from *S*_1_ to *S*_2_, and *d_o_* the distance from either to *C*. Equidistant *S*_1_, *S*_2_ and *S*_3_ make *T* equilateral, with a ratio $d_{o}/d_{s}=1/\sqrt{3}\approx 0.58$: deviation from this indicates a less optimal triangle. CRA uses sensor distances to circumcircle centers as a stand-in for those to every point in the field from its nearest sensor.

**Figure 5.**
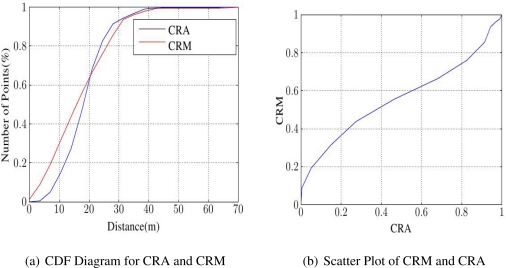
Comparing CRA with CRM.

For CRA, we propose these steps. First, find the neighbors by a DT algorithm. Next, calculate the average value of *d_o_/d_s_* for each triangle. These values are used as samples for the distances of all points of the field to their nearest sensors. The results show that the histogram of distribution of these sample points is very close to CRM (Figure 5). Moreover, it can be shown that the R-Square goodness of fit from CRA to true CRM is quite close to the perfect fit value 1. Table 1 is based on 1,000 random samples for each sensor density, in a 1,000 m × 1,000 m field.

### 4.4. Uniformity of Coverage

Uniformity of coverage was quantified in [28,29] by the Mean Relative Deviation (MRD) formula:.

(2) $$\mathrm{MRD}=\frac{\sum_{i=1}^{N}\left|\rho_{S_{i}}-\rho_{S}\right|}{N\cdot \rho_{S}},$$

where *ρ_S_* is the spatial density of the field and *ρ_S_i__* is the spatial density for the *i*^th^ portion of the field. The sets *S_i_* and *S_j_* for *i* ≠ *j* may intersect, and a bigger intersection makes Equation 2 more precise. We call this the window effect. Moreover, increasing the number *N* of samples leads to more accurate results. However, by [28,29], increasing *N* and window size add dramatically to computational complexity.

To explore the window effect on Equation 2, we studied a 1,000 m × 1,000 m field with 600 randomly placed sensors. The window size is from 100 m × 100 m to the maximum 1,000 m × 1,000 m, and each step moves the window 1 m in *x* or *y*, for all possible *N* = 1,000,000 locations in the field.

**Figure 6.**
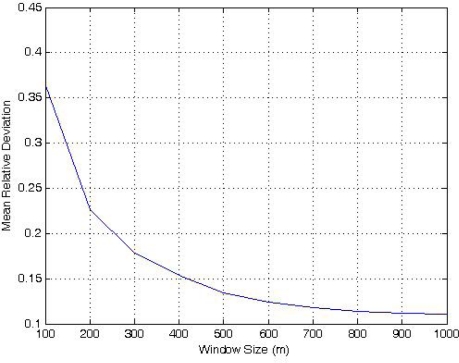
The effect of window size on MRD.

As shown in Figure 6, window size has a significant effect in Equation 2, so it is not wise to reduce it to save computation. To compare this method with our proposed algorithm, we show that even with the maximum window size (the whole field), Equation 2 is less precise than our method.

We define Uniformity of Coverage (UoC) as the relative standard deviation of the length of lines connected to each vertex (sensor).

(3) $$\mathrm{UoC}=\sigma_{s}/\bar{s}$$

(4) $$s_{i}=\frac{\sum l_{i,k}}{n_{k}}$$

where *l_i,k_* is the distance between connected sensors *i* and *k*, and *n_k_* is the number of lines.

Smaller is better for both UoC and MRD, and zero means absolute uniformity. The best known uniform distributions are grid and hexagonal deployments. For hexagonal deployment UoC is zero, as every line connected to each sensor has the same length and so their standard deviation is zero. To compare UoC and MRD we choose a random deployment and a grid deployment of 600 sensors in a 1,000 m × 1,000 m field, and apply two different changes to test the sensitivity of the algorithms. First, one sensor is deleted to see any changes in UoC and MRD. Then, one sensor is moved, to test the sensitivity of each algorithm to small movements.

As Table 2 shows, MRD detected no change in uniformity value in either grid and random deployment when the sensor moved about 1*m*. The UoC increase detects that moving a sensor in the grid deployment reduces uniformity. Moreover, one would expect a grid deployment to have higher uniformity value than a random one, but the MRD results do not reflect that: for random deployment the MRD is 0.1105, and for grid deployment 0.1168. Again, one expects removing a sensor from a grid deployment to reduce uniformity, but the MRD result is the opposite.

Thus, UoC is more sensitive and more accurate for measuring the uniformity of coverage than the MRD measure proposed in [28,29].

### 4.5. Largest Empty Circle

One feature of an optimized triangulation is that no vertex *v* is inside the circumcircle of any triangle whose corners do not include *v*. So, the circumcircle of a DT triangle is the largest empty space among three vertices, or in other words, three sensors. This concept has already been used by [20], as the Largest Empty Circle (LEC), to determine the best position for the next sensor deployment. Here, we propose this property to find the largest uncovered area in a WSN.

To find the LEC of a WSN in a field *F*, first find the circumcircle of every triangle in *F*, then trim those which extend outside *F*. The biggest area among all circles is the LEC, a good benchmark to compare QoC among different applications. Its radius shows the deepest point in the field, farthest from the nearest sensors. If this value is lower than the sensing coverage, an application knows for sure that it fully covers the mission field.

**Figure 7.**
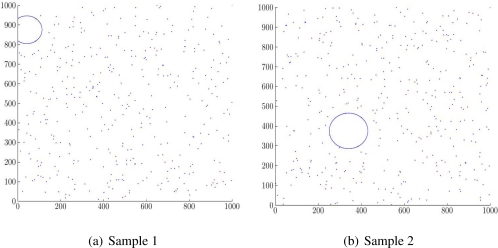
LEC Position And Size in Sample 1 And Sample 2.

Figure 7 shows two scenarios with very close circular coverage, but unequal LEC value.

## 5. Conclusions

Proper information about the coverage in a Wireless Sensor Network could have high impact on the algorithms designed to provide it. Older coverage measurement tools just provide a simple ratio of covered to desired area. Finding the shape of the coverage on the field could help researchers to create more uniform coverage and to prolong the network lifetime. In this paper, we have proposed a new measurement scheme, based on DT, which gives detailed information about the areas between sensors, distance between sensors, and fat, healthy and thin sensors. This information can improve understanding of the coverage properties of different coverage promising algorithms, and comparison among them. This work is funded by Fundamental Research Grant Scheme (FRGS) under project number 78458.

## Figures and Tables

**Table 1.** The Goodness of Fit for CRM.

Sensor No.	R-Square Mean	R-Square Variance
100	0.9383	0.0016
200	0.9416	0.0018
300	0.9386	0.0015
400	0.9391	0.0019
500	0.9332	0.0014
600	0.9345	0.0012
700	0.9251	0.0022
800	0.9201	0.0025
900	0.9098	0.0026
1000	0.8977	0.0027

**Table 2.** Comparing UoC and MRD methods.

	Random Deployment		Grid Deployment	

	UoC	MRD	UoC	MRD
Normal	0.6325	0.1105	0.0644	0.1168
Movement (1 m)	0.6324	0.1105	0.0646	0.1168
Movement (10 m)	0.6321	0.1104	0.0650	0.1168
Removing one sensor	0.6321	0.1101	0.0654	0.1160
